# Cultural shifts: an examination of cervical cancer stigma across age groups in the Caribbean

**DOI:** 10.1093/jncics/pkae075

**Published:** 2024-09-05

**Authors:** Gaole Song, Kamilah Thomas-Purcell, Diadrey-Anne Sealy, Althea Bailey, Camille Ragin, Kimlin Ashing

**Affiliations:** Department of Population Sciences, City of Hope Comprehensive Cancer Center, Duarte, CA, USA; Department of Health Science, Nova Southeastern University, Ft. Lauderdale, FL, USA; School of Public Health, Loma Linda University, Loma Linda, CA, USA; Department of Community Health and Psychiatry, University of the West Indies, Mona, Jamaica; Cancer Prevention and Control Program, Fox Chase Cancer Center – Temple University Health System, Philadelphia, PA, USA; African Caribbean Cancer Consortium, Philadelphia, PA, USA; Department of Population Sciences, City of Hope Comprehensive Cancer Center, Duarte, CA, USA; African Caribbean Cancer Consortium, Philadelphia, PA, USA

## Abstract

**Background:**

Cervical cancer-related stigma is common but understudied in the Caribbean. This study aims to describe the age difference of cervical cancer stigma and to evaluate the influence on the prevention practices among the Caribbean nonpatient population in Jamaica, Grenada, and Trinidad and Tobago.

**Methods:**

A cross-sectional study involving 1209 participants was conducted using a culturally trans-created Cancer Stigma Scale for the Caribbean context and supplemented with questions on cervical cancer and human papillomavirus (HPV) and HPV vaccine knowledge and beliefs. Descriptive analyses and χ^2^ tests were conducted.

**Results:**

The χ^2^ tests showed age is statistically significantly related to participants’ response to stigma items such as “community members believe cervical cancer is viewed as shameful” (*P = *.0001); “women with cervical cancer are treated with less respect than usual by others” (*P < *.0001); “women with cervical cancer are rejected by family members” (*P = *.0007); “women with cervical cancer are rejected by intimate partners” (*P < *.0001); and “intimate partners blame women for having cervical cancer” (*P = *.0032). Additionally, age has statistically significant associations with endorsements of negative views of cervical cancer from the community (*P < *.0001) and family (*P < *.0001) as key barriers to cervical cancer care (item: “discourage women from seeking and obtaining screening and treatment”). Notably, younger respondents (18-25 years) are more sensitized to the unfair stigma and hold more stigma.

**Conclusions:**

Among Caribbeans, age influences cervical cancer stigma. Younger persons acknowledged greater stigma within families and communities. This study can guide age-informed interventions and programs to reduce stigma and improve cervical cancer screening and care seeking to reduce cervical cancer burden and disparities.

Age has an important influence on health beliefs and practices. Various studies show age influences lifestyle, health behaviors, treatment responses, reactions, beliefs, and practices (eg, stigma) associated with illnesses (1-5). Cancer, in particular cervical cancer, remains one of the most stigmatized cancers or illnesses. Compared with women living in developed countries, women who live in developing countries are more vulnerable to cervical cancer due to the existing inequities in wealth, sex, and access to health services (6,7). In the Americas, an estimated 35 700 deaths are caused by cervical cancer each year, and the majority (80%) of these patients are in Latin America and the Caribbean (8). If current trends continue by 2030, deaths caused by cervical cancer are predicted to increase to more than 51 500, and nearly 90% of these deaths will occur in Latin America and the Caribbean (8). Therefore, factors that affect cervical cancer prevention, including stigma and demographic characteristics that influence stigma, must be prioritized if the reduction of premature death and disability caused by cervical cancer is to be realized.

Cervical cancer is indeed preventable. It is highly preventable and treatable, with up to 93% of patients with cervical cancer being avoidable through effective screening and human papillomavirus (HPV) vaccination (9). Getting vaccinated against HPV and having regular screening tests, such as the Pap test (or Pap smear) and the HPV test against the most viral HPV infections, can provide robust protection for children and young adults, and even middle-aged individuals (10,11). By implementing these prevention measures comprehensively and widely, we have the potential to significantly reduce the incidence and mortality of cervical cancer and save thousands of lives.

However, despite the high burden and available prevention services, cervical cancer screening rates are desperately low in the Caribbean. One reason for insufficient cervical cancer prevention and early detection is cancer stigma, which influences the uptake of preventive services in the Caribbean (12,13). Cancer stigma is the negative attitude of society toward cancer, and it is influenced by income, ethnicity, gender norms, culture, and type of cancer (14,15). Women experience additional stigma when cancers (such as cervical cancer) are linked to their reproductive system and traditional gender roles (14).

Age is likely to influence both health-related behavior and health beliefs (1-5). Yet, the important age characteristic is generally lacking in cervical cancer stigma research. Previous studies explored how the overall stigma may affect health outcomes (12-15); however, little is understood about age influences and differences in health-related stigma. It is also unclear whether individuals in different age groups perceive stigma differently, especially a lack of research focusing on investigating associations between age and cervical cancer stigma in the Caribbean. Therefore, to bridge the research gap, this study aimed to evaluate age differences in cervical cancer stigma among nonpatient populations in Jamaica, Grenada, and Trinidad and Tobago because these countries have been found by the Pan American Health Organization to have higher incidence rates of cervical cancer in the non-Latin Caribbean and to share similar cultural and social contexts (16).

## Methods

### Study design and settings

This study applied a community-based participatory research (CBPR) approach, which is a patient-centered, community-driven research approach to address health disparities and has been widely used in community-related public health research such as this study (17). Grenada, Jamaica, and Trinidad and Tobago, among the many Caribbean nations characterized by low rates of Pap testing and a high incidence of cervical cancer, served as foundational locations for this multinational study. All investigators collaborated closely with a multisectoral community advisory council and community leaders in each country to develop recruitment materials and to conduct a cross-sectional quantitative study. This study has been reviewed and approved by the City of Hope Comprehensive Cancer Center Institutional Review Board (IRB), and IRB approvals were also obtained in each country to conduct this study.

### Participants

A total of 2292 participants responded to the survey. After excluding participants with incomplete responses, this study included 1209 participants who were aged 18 to 85 and who lived in Jamaica, Trinidad and Tobago, and Grenada for at least 10 years (completion rate: 52.8%, no missing data in the used dataset). All participants were English proficient and provided written consent before answering any questions. A convenience sampling method was applied. Participants were recruited through printed study flyers posted in commercial establishments including grocery and retail outlets and electronic study flyers posted and advertised via various social media platforms, including Facebook, Instagram, and WhatsApp. Additionally, investigators collaborated with local hospitals, family practices, and primary care clinics on each island and delivered a WhatsApp or email message with the study flyer and link to the study to the participants recruited from their facilities.

### Data collection

Data collection took place online from October 2022 to March 2023. Participants provided their self-reported responses through the Research Electronic Data Capture system (REDCap), which is a secure, web-based application used for a wide variety of research studies.

### Measures

The survey used in this study was the culturally trans-created Cancer Stigma Scale (CASS) for the Caribbean context and supplemented with questions on cervical cancer and HPV and HPV vaccine knowledge and beliefs (12,13,18). Investigators transcreated the existing validated CASS following a qualitative approach consisting of focus groups held with key informants and stakeholders to provide input on cultural and contextual changes to CASS and ideas/content/domains for additional items for the culturally transcreated scale (19). The transcreated scale was discussed and pilot-tested with a community advisory group representing cancer societies, the Planned Parenthood Association, health advocacy groups, and community members. Self-reported demographic information (including age range, sex, sexual orientation, educational level, income range, employment status, and relationship status) was collected. Cervical cancer stigma was measured using the updated scale from 4 subscales, including the cancer stigma subscale, the social impact subscale, the health beliefs subscale, and the quality of care subscale. Based on CASS, there are 6 domains to be measured in our culturally trans-created scale:

1. Awkwardness: questions assessed whether people feel comfortable around women with cervical cancer (eg, “employer/coworkers discriminate against persons with cervical cancer”)
2. Severity: questions assessed how people view the severity of diagnosing cervical cancer (eg, “once you’ve had cancer, you can never be ‘normal’ again”)
3. Avoidance: questions measured whether people would keep their distance from women with cervical cancer (eg, “I would feel comfortable around someone with cancer”)
4. Personal responsibility: questions measured whether people believe an individual’s actions are considered to have contributed to their cervical cancer (eg, “a person with cancer should be blamed for their condition”)
5. Policy opposition: questions measured how people think the government and the public should take action toward the care and treatment of cancer patients (eg, “more government funding should be spent on the care and treatment of those with cancer”)
6. Financial discrimination: questions assessed how people think about cancer patients benefiting from bank and insurance services (eg, “banks should be allowed to refuse mortgage applications for cancer-related reasons”)

To better understand the cultural and social influence on cancer stigma, the questionnaire also included questions such as “In my community and culture, women with cervical cancer are looked down on,” “My culture believes that women with cervical cancer deserve less respect,” “Intimate partners reject women with cervical cancer,” etc.

In the cancer stigma subscale, we assessed participants’ belief in cervical cancer on a 5-point Likert scale (“strongly agree,” “agree,” “not sure,” “disagree,” “strongly disagree”). For the social impact subscale and health beliefs subscale, participants’ responses were recorded on a 5-point Likert scale (“strongly agree,” “agree,” “neither agree nor disagree,” “disagree,” “strongly disagree”).

This study combined “strongly agree” and “agree” to better describe the cancer stigma in different age groups.

### Data analysis

Incomplete responses were not considered in this analysis. Categorical data were reported in frequency and percentage. A cancer stigma variable was created. Cancer stigma responses were found to be not normally distributed. The normality test gave a significance of less than 0.001. Comparisons of skewness and kurtosis gave values greater than 1.96 and/or less than -1.96. To investigate the associations between age groups and cervical cancer stigma items, χ^2^ tests were performed. No other statistical tests were used to explore relationships between different variables. A *P* value of less than .05 as the test of statistical significance was set. All statistical analysis was performed using SAS 9.4 (20).

## Results

### Demographic results

Among all participants, 48.8% of respondents resided in Jamaica, 26.2% in Grenada, and 23.8% in Trinidad and Tobago. In total, 78.1% of participants were aged between 18 and 45 years, and most respondents were female (81.5%). Many survey participants had a university-level education (44.1%), with a further 52.2% having a secondary, community college, or trade and/or technical school level of education. More than half of respondents were employed on a full-time basis (53.0%), with 17.0% being students. Slightly more than half (51.0%) were single, and most participants were heterosexual or straight (88.7%). The demographic characteristics stratified by age groups of the participants are summarized in Table 1.

**Table 1.** Demographic characteristics of participants by age groups (Jamaica, Trinidad & Tobago, Grenada, 2023, N = 1209**)**

	Age group						Total
	Unweighted number of sample (n, %)						N = 1209
Characteristics	18-25	26-35	36-45	46-55	56-65	66+	
	n = 403	n = 282	n = 259	n = 129	n = 92	n = 44	
Residence country							
Jamaica	219 (54.3%)	148 (52.5%)	102 (39.4%)	55 (42.6%)	45 (48.9%)	21 (47.7%)	590 (48.8%)
Trinidad and Tobago	27 (6.7%)	60 (21.3%)	92 (35.5%)	58 (45.0%)	29 (31.5%)	22 (50.0%)	288 (23.8%)
Grenada	153 (38.0%)	71 (25.2%)	60 (23.2%)	16 (12.4%)	16 (17.4%)	1 (2.3%)	317 (26.2%)
Other	4 (1.0%)	3 (1.1%)	5 (1.9%)	0	2 (2.2%)	0	14 (1.2%)
Gender							
Male	97 (24.1%)	62 (22.0%)	26 (10.0%)	10 (7.8%)	13 (14.1%)	5 (11.4%)	213 (17.6%)
Female	304 (75.4%)	218 (77.3%)	232 (89.6%)	114 (88.4%)	78 (84.8%)	39 (88.6%)	985 (81.5%)
Transgender	0	1 (0.4%)	0	2 (1.6%)	0	0	3 (0.2%)
Other	1 (0.3%)	0	0	0	0	0	1 (0.1%)
Prefer not to answer	1 (0.3%)	1 (0.4%)	1 (0.4%)	3 (2.3%)	1 (1.1%)	0	7 (0.6%)
Sexual orientation							
Heterosexual/straight	345 (85.6%)	255 (90.4%)	237 (91.5%)	109 (84.5%)	88 (95.7%)	38 (86.4%)	1072 (88.7%)
Men who have sex with men (MSM)	0	0	0	0	1 (1.1%)	0	1 (0.1%)
Lesbian	3 (0.7%)	1 (0.4%)	0	1 (0.8%)	0	0	4 (0.3%)
Bisexual	30 (7.4%)	9 (3.2%)	8 (3.1%)	1 (0.8%)	0	1 (2.3%)	49 (4.1%)
Other	8 (2.0%)	5 (1.8%)	5 (1.9%)	5 (3.9%)	1 (1.1%)	1 (2.3%)	25 (2.1%)
Prefer not to answer	17 (4.2%)	12(4.3%)	9 (3.5%)	13 (10.1%)	2 (2.2%)	4 (9.1%)	57 (4.7%)
Relationship status							
Currently married/living with someone	45 (11.2%)	110 (39.0%)	130 (50.2%)	63 (48.8%)	45 (48.9%)	23 (52.3%)	416 (34.4%)
Divorced/lived with someone in the past	6 (1.5%)	12 (4.3%)	21 (8.1%)	15 (11.6%)	18 (19.6%)	12 (27.3%)	84 (6.9%)
Single	299 (74.2%)	146 (51.8%)	95 (36.7%)	45 (34.9%)	25 (27.2%)	7 (15.9%)	617 (51.0%)
Prefer not to answer	53 (13.2%)	14 (5.0%)	13 (5.0%)	6 (4.7%)	4 (4.4%)	2 (4.6%)	92 (7.6%)
Educational level							
Primary	1 (0.3%)	0	1 (0.4%)	5 (3.9%)	0	5 (11.4%)	12 (1.0%)
Secondary and/or high	133 (33.0%)	53 (18.8%)	47 (18.2%)	27 (20.9%)	22 (23.9%)	8 (18.2%)	290 (24.0%)
Community college	116 (28.8%)	53 (18.8%)	34 (13.1%)	17 (13.2%)	10 (10.9%)	2 (4.6%)	232 (19.2%)
Trade or technical school	44 (10.9%)	25 (8.9%)	17 (6.6%)	12 (9.3%)	6 (6.5%)	5 (11.4%)	109 (9.0%)
University	96 (23.8%)	148 (52.5%)	154 (59.5%)	67 (51.9%)	49 (53.3%)	19 (43.2%)	533 (44.1%)
Prefer not to answer	13 (3.2%)	3 (1.1%)	6 (2.3%)	1 (0.8%)	5 (5.4%)	5 (11.4%)	33 (2.7%)
Employment status							
Full-time	126 (31.3%)	183 (64.9%)	190 (73.4%)	93 (72.1%)	45 (48.9%)	4 (9.1%)	641 (53.0%)
Part-time	36 (8.9%)	40 (14.2%)	26 (10.0%)	17 (13.2%)	6 (6.5%)	0	125 (10.3%)
Student	172 (42.7%)	23 (8.2%)	7 (2.7%)	3 (2.3%)	0	0	205 (17.0%)
Retired	0	0	0	1 (0.8%)	32 (34.8%)	37 (84.1%)	70 (5.8%)
Not employed	58 (14.4%)	30 (10.6%)	25 (9.7%)	10 (7.8%)	6 (6.5%)	1 (2.3%)	130 (10.8%)
Prefer not to answer	11 (2.7%)	6 (2.1%)	11 (4.3%)	5 (3.9%)	3 (3.3%)	2 (4.6%)	38 (3.1%)

### Age difference in cervical cancer stigma

Response percentages stratified by age groups are shown in Table 2.

**Table 2.** Age difference in cancer stigma (Jamaica, Trinidad and Tobago, Grenada, 2023; N = 1209)

Age group (unweighted n, % of Strongly Agree and Agree)							
Cervical cancer stigma item	18-25	26-35	36-45	46-55	56-65	66+	*P* value
	n = 403	n = 282	n = 259	n = 129	n = 92	n = 44	
Women with cervical cancer are treated with less respect than usual by others	92 (22.8%)	39 (13.8%)	32 (12.4%)	16 (12.4%)	16 (10.9%)	4 (9.1%)	<.0001
Women are concerned they could “catch” cervical cancer via contact like a handshake	27 (6.7%)	23 (8.2%)	10 (3.9%)	2 (1.6%)	9 (9.8%)	1 (2.3%)	.0263
Women with cervical cancer must keep it a secret	29 (7.2%)	32 (11.4%)	23 (8.9%)	8 (6.2%)	10 (10.9%)	1 (2.3%)	.0352
Women with cervical cancer are rejected by family members	36 (8.9%)	25 (8.9%)	18 (7.0%)	8 (6.2%)	10 (10.9%)	4 (9.1%)	.0007
Women with cervical cancer are rejected by intimate partners	130 (32.3%)	69 (24.5%)	48 (18.5%)	30 (23.6%)	21 (22.8%)	13 (29.5%)	<.0001
Intimate partners blame women for having cervical cancer	100 (24.8%)	55 (19.5%)	37 (14.3%)	29 (22.5%)	18 (19.6%)	11 (25.0%)	.0032
My community’s negative view of cervical cancer may discourage women from getting the Pap smear	159 (39.5%)	78 (27.7%)	59 (22.8%)	33 (25.6%)	21 (22.8%)	7 (15.9%)	<.0001
My community’s negative view of cervical cancer may discourage women from getting further diagnosis after an abnormal Pap smear	141 (35.0%)	80 (28.4%)	63 (24.3%)	32 (24.8%)	22 (23.9%)	9 (20.5%)	<.0001
The fear of family reaction and rejection may discourage women from getting further diagnosis after an abnormal Pap smear	194 (48.1%)	93 (33.0%)	75 (29.0%)	30 (23.3%)	27 (29.4%)	10 (22.7%)	<.0001
Many women may have the type of human papillomavirus (HPV) that causes cervical cancer, but only women without regular medical care get cervical cancer	54 (13.4%)	52 (18.4%)	40 (15.4%)	24 (18.6%)	16 (17.4%)	4 (9.1%)	<.0001
It is unfair that society blames women with cervical cancer for their illness	296 (73.5%)	206 (73.1%)	172 (66.4%)	75 (58.1%)	56 (60.9%)	29 (65.9%)	.0005
In my community and culture, women with cervical cancer are looked down on	78 (19.4%)	40 (14.2%)	24 (9.3%)	23 (17.8%)	15 (16.3%)	8 (18.2%)	.0002
Women with cervical cancer are more isolated	131 (32.5%)	72 (25.5%)	55 (21.2%)	32 (24.8%)	18 (19.6%)	11 (25.0%)	<.0001
My family believes that women with cervical cancer deserve less respect	22 (5.5%)	17 (6.0%)	9 (3.5%)	11 (8.5%)	8 (8.7%)	0	.0120
My culture believes that women with cervical cancer deserve less respect	33 (8.2%)	22 (7.8%)	13 (5.0%)	6 (4.7%)	11 (12.0%)	3 (6.8%)	.0009
In my community, cervical cancer is viewed as shameful	43 (10.7%)	30 (10.6%)	17 (6.6%)	8 (6.2%)	8 (8.7%)	4 (9.1%)	.0001
Cervical cancer can be caused by hormones (birth control pills, hormone replacement therapy)	156 (38.7%)	97 (34.4%)	66 (25.5%)	31 (24.0%)	9 (9.8%)	5 (11.4%)	<.0001
Cervical cancer can be caused by personal/familial punishment for wrongdoing	27 (6.7%)	17 (6.0%)	10 (3.9%)	4 (3.1%)	1 (1.1%)	1 (2.3%)	<.0001
Cervical cancer can be caused by having an abortion	104 (25.8%)	59 (20.9%)	30 (11.6%)	9 (7.0%)	5 (5.4%)	1 (2.3%)	<.0001
Cervical cancer can be caused by being unclean (not washing often during menstrual period or after sexual intercourse)	118 (29.3%)	58 (20.6%)	30 (11.6%)	13 (10.1%)	9 (9.8%)	1 (2.3%)	<.0001
Cervical cancer can be caused by having sex with a partner or man with HPV (ie, human papillomavirus/genital warts)	237 (58.8%)	168 (59.6%)	160 (61.8%)	75 (58.1%)	53 (57.6%)	26 (59.1%)	.0302
Cervical cancer can be caused by a stressful life	81 (20.1%)	81 (28.7%)	77 (29.7%)	33 (25.6%)	15 (16.3%)	5 (11.4%)	<.0001

### Community and society stigma

In this domain, questions were asked to measure how community, society, and culture would influence people’s attitudes and feelings toward patients with cervical cancer.

Particularly, χ^2^ tests showed age is statistically significantly related to respondents’ opinions of items such as “Women with cervical cancer are treated with less respect than usual by others (*P < *.0001)” and “Women with cervical cancer must keep it a secret (*P = *.0352).” In addition, age is statistically significantly associated with community stigma, including “My community’s negative view of cervical cancer may discourage women from getting the Pap smear (*P < *.0001),” “My community’s negative view of cervical cancer may discourage women from getting further diagnosis after an abnormal Pap smear (*P < *.0001),” “In my community and culture, women with cervical cancer are looked down on (*P = *.0002),” and “In my community, cervical cancer is viewed as shameful (*P = *.0001).” Age and culture also interact: “My culture believes that women with cervical cancer deserve less respect (*P = *.0009).” Meanwhile, age as a factor showed significant relations with society stigma items such as “Women with cervical cancer are more isolated (*P < *.0001)” and “It is unfair that society blames women with cervical cancer for their illness (*P = *.0005).”

Notably, compared with participants who are aged more than 35, younger (18-25 or 26-35 years old) participants reported more serious stigma than others for some items. For example, 11.4% of respondents aged 26 to 35 agreed that women with cervical cancer must keep it a secret, which was the highest percentage. Also, respondents aged 18 to 25 reported the highest percentages of community-related stigma: 22.8% of them agreed that women with cervical cancer are treated with less respect than usual by others; 39.5% of them believed the community’s negative view of cervical cancer may discourage women from getting the Pap smear, and 35.0% of them agreed with the opinion that community’s negative view of cervical cancer may discourage women from getting further diagnosis after an abnormal Pap smear. Meanwhile, almost 1 in 5 participants aged 18 to 25 believed that in their community and culture, women with cervical cancer are looked down on; more than 1 in 10 participants aged 18 to 25 believed that in their community cervical cancer is viewed as shameful. Additionally, 32.51% of participants aged 18 to 25 agreed that women with cervical cancer are more isolated.

### Interpersonal relationship stigma

In this domain, questions were asked to measure people’s attitudes and feelings toward patients with cervical cancer in familial, intimate, and other interpersonal relationships.

The χ^2^ tests also showed age is statistically significantly related to respondents’ opinions of items such as “Intimate partners blame women for having cervical cancer (*P = *.0032)” and “Women with cervical cancer are rejected by intimate partners (*P < *.0001).” In addition, age is statistically significantly associated with family stigma, such as “Women with cervical cancer are rejected by family members (*P = *.0007),” “The fear of family reaction and rejection may discourage women from getting further diagnosis after an abnormal Pap smear (*P < *.0001),” and “My family believes that women with cervical cancer deserve less respect (*P = *.0120).”

In this domain, younger participants also reported more stigma for some items: more than 30% of respondents aged 18 to 25 believed women with cervical cancer are rejected by intimate partners, and almost half of them agreed that the fear of family reaction and rejection may discourage women from getting further diagnosis after an abnormal Pap smear.

### Age and cervical cancer knowledge

In this domain, questions were asked to measure people’s knowledge of cervical cancer causes, risk factors, and prevention. The χ^2^ tests indicated age was statistically significantly associated with items including “Women are concerned they could ‘catch’ cervical cancer via contact like a handshake (*P = *.0263),” “Many women may have the type of HPV that causes cervical cancer, but only women without regular medical care get cervical cancer (*P < *.0001),” “Cervical cancer can be caused by hormones (birth control pills, hormone replacement therapy) (*P < *.0001),” “Cervical cancer can be caused by personal/familial punishment for wrongdoing (*P < *.0001),” “Cervical cancer can be caused by having an abortion (*P < *.0001),” “Cervical cancer can be caused by being unclean (not washing often during the menstrual period or after sexual intercourse) (*P < *.0001),” “Cervical cancer can be caused by a stressful life (*P < *.0001),” and “Cervical cancer can be caused by having sex with a partner or man with HPV (ie, human papillomavirus/genital warts) (*P = *.0302).”

Notably, more younger participants (18 to 35) agreed that “Cervical cancer can be caused by hormones (birth control pills, hormone replacement therapy)”—73.1%, “Cervical cancer can be caused by personal/familial punishment for wrongdoing”—12.7%, “Cervical cancer can be caused by having an abortion”—46.7%, and “Cervical cancer can be caused by being unclean (not washing often during the menstrual period or after sexual intercourse)”—49.9%.

## Discussion

This is one of the first studies examining age differences in cancer stigma within the Caribbean region. Previous literature focused more on cervical cancer incidence and prevalence changes in this region but did not explore potential contributing factors. Age is an important factor in health behaviors and health beliefs; cancer stigma is closely related to an individual’s health beliefs. How age affects cancer stigma is understudied; there is especially a lack of research focusing on the intersections of age and cervical cancer-related stigma, prevention, and screening. This study reinforces the existing literature that age as a demographic characteristic may constitute cultural groupings that influence dimensions of disease-related and cancer stigma stemming from community, familial, and intimate relations. The findings of this study indicated age is significantly related to cervical cancer stigma. Younger participants agreed that women with cervical cancer are treated with less respect than usual by others in their community. One explanation for this stigma is the fact that HPV is sexually transmitted and causes cervical cancer and that younger persons may be more sensitized to societally dictated and endorsed stigma (21-24). The results of this study also found that younger respondents reported persistent community, familial, and intimate relation stigma associated with cervical cancer, although they do not have a strong personal stigma related to cervical cancer. Younger participants held a stronger belief in the stigma itself (eg, believing a community views cervical cancer as shameful) and a heightened sensitivity to the potential negative consequences of the stigma (eg, being ostracized by family). Cancer stigma was associated with a low likelihood that women with cervical cancer would seek support from their family members and intimate partners (25). Previous research has stated that social support is positively associated with preventive practices for cervical cancer (26).

Most participants, across all age groups, noted that stigma exists in their community and society. This unique study underscored that there is limited research addressing the impact of stigma on cervical cancer prevention, including Pap testing and HPV vaccination attitude and uptake in the Caribbean region. Previous research indicated strategies such as reminders and advice from clinicians can significantly increase colorectal cancer screening (27,28). Thus, similarly, providers ought to be a part of the solution to address stigma and increase cervical screening and prevention by actively encouraging patients to undergo cervical cancer screening and HPV vaccination. They should also promote education that involves and targets diverse age groups.

Interventions must consider tailoring to age categories and groups to improve the efficacy and effectiveness of strategies to reduce stigma and improve preventive practice. Additionally, a previous study suggested increasing awareness of the disease, treatment steps, and possible complications would reduce stigma related to the disease (29). Therefore, a possible solution to eliminate stigma is that public awareness interventions should highlight the treatability and potential curability of cervical cancer when detected early. Educational interventions can take advantage of social media as an effective method for intervention dissemination, which can reach a younger population. Social media campaigns can also emphasize that cervical cancer is highly treatable and even curable when detected at early stages. The efficacy and effectiveness of media in health behavior change have been well documented, especially with Black and Afro-Caribbean populations in cancer (30,31). Our previous pilot study also showed social marketing intervention can be an effective method to reduce stigma and increase cervical cancer screening (32).

To enhance prevention, screening, diagnosis, treatment, and survivorship care for cervical cancer, it is crucial to address and challenge the societal and social stigma, ie, isolation, rejection, and personal stigma such as shame.

### Limitations

This study has several limitations. First, the majority of our respondents were younger and women, which may influence the findings. To obtain greater participant diversity, it is essential to include a larger representation of men and older persons in the study. A significant portion of the data was collected online, potentially biasing the sample toward a more educated population. In addition, participants in this study tended to be younger, especially given that the survey was conducted electronically. Future studies will recruit more diverse participants and include other Caribbean countries. Additionally, we also will make the stigma-related survey themes more comprehensive to include questions such as measuring participants’ opinions on awareness and preference of strategies that can help reduce stigma in their communities and cultures, whether they believe HPV infection can cause stigma, etc. These questions will be beneficial to intervention design. Despite these limitations, to our knowledge, this first-of-its-kind study offers new knowledge about the influence of age on cervical cancer stigma. Our results have the potential to guide and inform future research and practice.

## Data Availability

Research data are not shared due to existing consent and Institutional Review Board constraints.
